# Human type 2 macrophages biologically active soluble products in the editing of stress-induced depressive-like behavior

**DOI:** 10.1192/j.eurpsy.2021.2023

**Published:** 2021-08-13

**Authors:** E. Markova, E. Shevela, M. Knyazheva, I. Savkin, T. Amstislavskaya, A. Ostanin, E. Chernykh

**Affiliations:** 1 Neuroimmunology Lab, State Research Institute of Fundamental and Clinical Immunology, Novosibirsk, Russian Federation; 2 Cellular Immunotherapy Laboratory, State Research Institute of Fundamental and Clinical Immunology, Novosibirsk, Russian Federation; 3 Translational Biopsychiatry Lab, Research Institute of Neurosciences and Medicine, Novosibirsk, Russian Federation

**Keywords:** anti-inflammatory macrophages, depressive-like behavior, cytokines

## Abstract

**Introduction:**

In the scientific world widely discussed phenomenon of “cytokine-induced depression”. Macrophages have high plasticity and are able to control the inflammatory response; in particular, anti-inflammatory type-2 macrophages have a pronounced potential due to complex soluble factors production.

**Objectives:**

We have developed an original method for the type-2 macrophages generation; the resulting macrophages are characterized by the high level of a whole range of neurotrophic, neuroprotective, proangiogenic and anti-inflammatory factors production. The aim of the study was to investigate effects of human type-2 macrophages soluble products on behavioral phenotype and brain cytokines synthesis in depressive-like animals.

**Methods:**

Type-2 macrophages were generated by culturing an adherent fraction of mononuclear cells with 50 ng/ml recombinant human GM-CSF in serum deprivation conditions for 7 days. (CBA x C57Bl/6)F1 depressive-like male mice, developed under the long-term social stress, were undergoing the human type-2 macrophages conditioned medium intranasal administration (60 ml twice daily for one animal) for 6 days. Mice behavioral phenotyping was carried out using an automatic registration system (Noldus Information Technology). Cytokines were determined by ELISA.

**Results:**

Depressive-like mice behavioral phenotyping after type-2 macrophages conditioned medium administration revealed anhedonia decrease, motor activity stimulation in the open field and forced swimming tests, anxiety reduction in elevated plus maze. Behavioral changes were recorded against the pro-inflammatory cytokines (TNF-α, IL-1β, IL- 6, INFγ) decrease in striatum and hippocampus, as well as anti-inflammatory IL- 10 increase in hippocampus and hypothalamus.

**Conclusions:**

Results demonstrated the effectiveness of human type-2 macrophages biologically active soluble products in relation to the stress-induced depressive-like behavior editing

**Disclosure:**

No significant relationships.

